# Effect of siddha varmam therapy in the management of cervical spondylosis - A case series

**DOI:** 10.6026/973206300210199

**Published:** 2025-02-28

**Authors:** Manjari Venkatraman, Kalaivanan Karuppan, Nithyashree Murugappa, Harishma Karthikeyan, Abarna Balasubramani

**Affiliations:** 1 Department of Nanju Maruthuvam, National Institute of Siddha, Chennai - 600047, Tamil Nadu, India; 2 Department of Siddha Medicine, Government Primary Health Centre, Tiruvannamalai (Dt) - 604402, Tamil Nadu, India; 3 Department of Siddha Medicine , BSMS - II Professional, Nandha Siddha Medical College and Hospital, Pitchandipalayam, Erode - 638052, Tamil Nadu, India

**Keywords:** Cervical spondylosis, Ceganavatham, pain, siddha medicine, varmam therapy

## Abstract

Cervical spondylosis is a degenerative condition affecting the intervertebral discs and vertebral bodies of the neck, causing pain, stiffness and neurological symptoms. Cervical spondylosis is closely associated with Ceganavatham, where pain management is the primary treatment focus in Siddha medicine. Varmam therapy is a traditional Siddha technique that was applied to 10 patients with Ceganavatham using selected pressure points once every two days for 12 sessions. Pain and disability levels were measured using the Numeric Pain Rating Scale (NPRS) and Neck Disability Index (NDI) before, during and after treatment. The results showed a significant reduction in pain and disability, suggesting that Varmam therapy is an effective treatment for Ceganavatham. It should be noted that further studies are needed for validation.

## Background:

The term "cervical spondylosis" refers to a broad range of progressive degenerative alterations that impact every cervical spine component. Age-related deterioration of cervical spondylosis is common and occurs across multiple interspaces. It can lead to disk herniation, bone spur formation, spinal cord compression, or cervical spondylotic myelopathy. Two-thirds of the populations have neck pain at some time in their lives. Mechanical factors like neck strain, sports, & occupational, *etc.*, are more prominent pain in the neck [1]. Cervical spondylosis has a negative impact on quality of life due to its high disease burden. Previous studies showed that age was the main risk factor and a contributor to the incidence of cervical spondylosis. Postural changes may also contribute to cervical pain. In Cervical spondylosis, Cervical pain, pain aggravated by movements, referred pain between the shoulder blade and upper limb, cervical stiffness, tingling and weakness in upper limbs are common. If pain persists on mechanical injury or old age osteophytic changes also occur [2]. These signs and symptoms are closely correlated to Ceganavatham based on Siddha literature. As per Siddha literature Yugi Vaithiya Sinthamani and Pararasa sekaram, Ceganavatham has the following symptoms, pain below the neck and lower back, pain in both upper limbs, heaviness, numbness, depression and giddiness, burning sensation in both eyes, constipation and pain felt like scorpion biting over the body [3, 4, 5]. Varmam is a manipulation therapy of Siddha that helps to treat pain in musculoskeletal disorders such as spondylosis, arthritis, *etc.* Therefore, it is of interest to determine the effectiveness of Varmam therapy in the pain management of Ceganavatham.

## Case report:

This case series included patients having signs and symptoms of Ceganavatham to visit the OPD of National Institute of Siddha, Tambaram and Government Primary Health Centre - Siddha OPD, Perungattur. Cases that were evaluated initially included the history, physical examination and severity of pain based on the Numeric Rating Scale and Neck Disability Index [6, 7]. Demographic data were collected including age, sex and personal and medical history with duration since the onset of the condition. Pre, Intermediate treatment and post-treatment were assessed by the Numeric Rating Scale and Neck Disability Index. All the demographic data including age, gender, occupation, past history, duration and vital signs of all the patients are described in Table 1.

## Case presentation:

## Case - 1:

A 69-year-old male came with complaints of pain in neck radiating to right upper limb. Difficulty to use right upper limb to do regular activities like wearing a shirt, *etc.* is monitored for 6 months.

## Clinical examination:

On examination, the patient presented tenderness in right shoulder, restricted movement in right Lateral rotation of the neck.

## Case - 2:

A 37-year-old female came with the complaints of pain in the neck radiating to left shoulder up to fingers, neck stiffness since 2 years. The patient previously took treatment for cervical spondylosis for 5 months even though the pain hasn't reduced.

## Clinical examination:

On examination, the patient presented tenderness in right shoulder with aggravating pain on left lateral rotation.

## Radiological Findings:

X- Ray findings of Cervical Spine AP and Lateral view showed minimal anterior end plate, osteophytes at C5- C6 vertebrae.

## Case - 3:

A 53 - year-old female came with the complaints of pain in the neck, pain radiating to right upper limb with restriction of movements of right upper limb since 6 months.

## Clinical examination:

On examination, tenderness in right shoulder with aggravated pain and restricted movement of right lateral rotation and flexion.

## Case - 4:

A 37 - year-old female came with the complaints of pain in the neck, pain radiating to both upper limb, pain aggravates on moving neck since 6 months.

## Clinical examination:

On examination, tenderness present in both shoulders, pain aggravates with restricted movement in right lateral rotation.

## Case - 5:

A 39 - year-old female came with the complaints of pain in the neck, Stiffness in the back of neck & shoulder, pain aggravates on sleep, pain presents as scorpion bite in the back of the neck and shoulder, pain present in occipital region, sleeplessness since 18 months.

## Clinical examination:

On examination, tenderness present in right shoulder, spasm present in left shoulder, pain aggravates with lateral rotation and flexion.

## Case - 6:

A 42-year-old female came with the complaints of pain in neck radiating to right shoulder up to right elbow, pain aggravates on rotating the neck, pain present in right ankle joint, swelling in right shoulder occasionally since 3 months.

## Clinical examination:

On examination, tenderness present in right shoulder, pain aggravates on right lateral rotation, flexion and extension causes severe pain in C6-C7 region.

## Case - 7:

A 43-year-old male has the complaints of pain in the back of the neck, stiffness present in both shoulders with numbness present in left upper limb, giddiness present on changing the position of the neck since 18 months.

## Clinical examination:

On examination, tenderness present in left shoulder with aggravated pain on left lateral rotation of the neck.

## Case - 8:

A 23-year-old male has the complaints of pain present in the neck, pain radiating to left upper limb up to fingers, pain aggravates on prolonged sitting, swelling present in the back of the neck since 5 months.

## Clinical examination:

On examination, tenderness present in cervical region, right shoulder slightly elevated than the left shoulder, pain aggravates on flexion. X-Ray findings of cervical spine AP and Lateral view showed no abnormalities.

## Case - 9:

A 36-year-old female having the complaints of pain present in the neck, pain radiating to right shoulder and elbow, heaviness of right upper limb, pain aggravates on studying and driving since 7 months.

## Clinical examination:

On examination, tenderness right shoulder, pain aggravates on right lateral flexion of the neck, weakness of right upper limb, warmth and spasm present in right shoulder and forearm. X-Ray findings of cervical spine AP and Lateral view showed loss of curvature, straightening of the spine due to muscle spasm & C5-C6 Disc space reduced.

## Case - 10:

A 25-year-old female came with the complaints of pain in neck radiating to left upper limb, headache present sometimes since 3 months.

## Clinical examination:

On examination, tenderness present in right shoulder, spasm in left shoulder, pain aggravates on flexion of the neck.

## Diagnostic assessment:

Numeric Rating Scale and Neck Disability Index form were used for recording the pain and disability.

## Treatment:

The following Varmam points were manipulated for 12 sessions in an interval of 2 days for all study patients. 10 seconds gap between each manipulation was used [8] (Table 2).

## Outcome measure:

A Numeric pain rating scale (NRI) is a uni-dimensional measure of pain intensity, used to compare the pain severity before and after treatment. NRI is more sensitive and easy to use in routine treatment [6]. The Neck Disability Index was used to evaluate the nature, severity and impact of each participant's cervical pain and Varmam therapy. The Neck Disability Index is a reliable scale to assess the severity of disability caused by neck pain of different musculoskeletal aetiology. The NDI is a self - report questionnaires for assessing the disability of the neck. Each question is rated on a scale from 1 to 4 with a total score ranging from 0 to 28; the higher the score, the more severe the cervical pain [7]. Data were collected at baseline, 6^th^ Session (mid of treatment) and 12^th^ session (End of the treatment). The timeline and observation of the NPR and NDI is portrayed in Table 3 and Table 4. Prognosis of disease before and after Varmam therapy assed by NPR and NDI mentioned in (Figure 1 - (see PDF) and Figure 2 - (see PDF)). Additionally, participants were asked to fill out an adverse event form if necessary. Participants also were asked to give their overall impressions about the therapy at the end of their study.

## Results:

After the twelve sessions of Varmam therapy, the intensity of pain in - cervical region, upper back, heaviness of neck and radiating pain reduced after in all patients. The range of movements in neck is improved and to do their daily activities with ease. Results of Pain Score and Neck Disability Index - before, in-between and after treatment are listed in Table 3, Table 4 and prognosis is mentioned in (Figure 1 - (see PDF), Figure 2 - (see PDF)).

## Discussion:

Non-Steroidal Anti-inflammatory drugs are mainstay of treatment for pain. The range of motion of affected joints is in general improved with physiotherapy and exercises [9]. Due to expensive of treatment patients are turn towards Siddha Medicine. The primary management of cervical spondylosis is to reduce pain and improve neck movement [10, 11]. In Siddha, Varmam is a non-invasive, manipulation therapy used primarily for acute conditions and chronic pain management [12]. It is effective to treat musculoskeletal and nervous disorder. There are various Varmam points mentioned in the Siddha literature which when stimulated appropriately will be helpful in the management of several diseases. In the human body panic energy flows in particular pathway and it is concentrated in certain points called as Varmam. To manipulate life energy through to stimulate selected Varmam points with appropriate amount of pressure the pranic energy is release and the pain is reduced and helps to relieve from the diseases or disease management. In this way Varmam therapy may help to relieve from pain in Ceganavatham. The exact mechanism of action of Varmam is still not identified. The mechanism of Varmam therapy may be analogues to acupressure therapy.

The pressure therapy is explained by complex neuro hormonal responses on stimulating points. It encompasses counter action between hypothalamic-pituitary-adreno cortical axis that leads to over production of cortisol and cause relaxation responses. GATE control Theory by Melzack and Wall which suggests the acupressure at definite points transmits impulses to the brain and uninterrupted impulses shut the neural GATES and slower down pain intensity [13]. This improves or strengthens the pain perception threshold of our body. The mechanism of the pain pathway would have been impacted by the pressure manipulation using the above-mentioned Varmam points. Varmam is an inexpensive and patient-focused procedure. In this case studies cervical spondylosis patients are treated with Varmam therapy. Ceganavatham diagnosed 3 males and 7 females with the age group of 20-70 years without any comorbidity were chosen in this study. This study included cases have a pain scale at the range of 6 - 9 (Moderate to Severe) with the NDI score ranging from 18 - 86 % before the Varmam therapy. After the treatment procedure of 12 sessions of Varmam therapy, there is a marked difference in the range of NRI pain scale (4 - 2) and NDI (6 - 24%) in all study cases. Pain in the cervical region and radiating pain became severe to no pain, moderate to mild and severe to moderate. Neck disability becomes, complete to no disability, mild & moderate, severe to moderate & mild disability, moderate to mild and No disability. Thus, cervical spondylosis can be effectively treated with Varmam therapy. No patients discontinued the treatment procedure. The patients were further followed up for 2 months and found no recurrence of pain.

## Conclusion:

Data shows that Varmam therapy can be effectively used in the treatment of Ceganavatham (cervical spondylosis) to reduce pain and improve restricted movement. The mechanism of action and effect of the Siddha Varmam therapy in Ceganavatham (Cervical spondylosis) require randomised clinical trials.

## Patient perspectives:

The patients reported that they were highly satisfied with the treatment as they had a considerable reduction in pain. Moreover, the quality of life improved and there is no recurrence of pain.

## Informed consent:

Written informed consent was obtained from all the patients.

## Author contribution:

Manjari Venkatraman did the choice of Varmam treatment selection, literature review, case study, analysis and interpretation of data and formatting. Kalaivanan Karuppan and Harishma Karthikeyan did the case study. Abarna Balasubramani & Nithyashree Murugappa did the literature review and report Writing.

## Figures and Tables

**Table 1:** General examination findings with past history of study participants

**Case series**	**Age/Sex**	**Occupation**	**Past history**	**Heart rate/min**	**Pulse rate/min**	**BP in mm of Hg**
Case 1	69/M	Farmer	No H/O DM, SHT, Trauma , H/O Undergone surgery for nasal septal deviation 30 years ago.	83	83	130/70
Case 2	37/F	Home maker	No H/O DM, SHT, Trauma	74	74	130/90
Case 3	53/F	Home maker	No H/O DM, SHT, Trauma	74	74	130/90
Case 4	37/F	Home maker	No H/O DM, SHT, Trauma	72	72	120/80
Case 5	39/F	Attender	No H/O DM, SHT, Trauma	72	72	120/80
Case 6	42/F	Teacher	No H/O DM, SHT, Trauma H/O COVID-19 positive 3 years ago.	74	74	130/80
Case 7	43/M	Doctor	No H/O DM, SHT, Trauma	72	72	120/80
Case 8	23/M	Student	No H/O DM, SHT, Trauma	86	88	120/70
Case 9	36/F	Working in IT	No H/O DM, SHT, Trauma	72	70	130/80
Case 10	25/F	Student	No H/O DM, SHT, Trauma	74	76	120/70
H/O: History of,
DM: Diabetes mellitus,
SHT: Systemic Hyper Tension.

**Table 2:** Varmam points, their anatomical location and procedure for the management of Ceganavatham [8]

**Varmam**	**Location**	
Mudichu Varmam	C7 Prominence	Using the middle 3 fingers manipulate clockwise and anticlockwise 3 times each
Kakkaittai Varmam	Supra clavicular fossa on both sides	Press and release the supra clavicular fossa from backside for 3 times using middle 3 fingers.
Kaichulikki Varmam	4 fingerbreaths from occipital region	Manipulate the Varmam by pressing and releasing for 3 times at both sides using thumb finger.
Chippi Varmam	Two fingerbreadths downward from the kaichulukki	Using the tip of middle 3 fingers move up and down with mild pressure.
Savvu Varmam	Four fingerbreadths distal from the shoulder	Press the Varmam by thumb finger with mild pressure.
Kavuli Varmam	Web area in between the thumb	Using the tip of middle 3 fingers press by pumping motion.
Manipandha Varmam	Middle of the wrist joint	Manipulate using the mid of the thumb with mild pressure.
Soodothari Varmam	radial aspect of the forearm	Manipulate using the mid of the thumb with mild pressure.
Melmannai Varmam	Upper end of the calf muscle	Press the Varmam by thumb finger with mild pressure.
Keelmannai Varmam	Lower end of the calf muscle	Press the Varmam by thumb finger with mild pressure.

**Table 3:** Pain score assessment by numeric pain rating scale in study participants

**Assessment**	**Pre-treatment (0^th^ day)**	**6^th^ session**	**12^th^ session**
Case 1	9	5	0
Case 2	8	5	2
Case 3	7	5	3
Case 4	6	4	2
Case 5	9	6	2
Case 6	6	4	0
Case 7	8	6	4
Case 8	6	5	3
Case 9	7	5	3
Case 10	7	6	4
0: No Pain,
1 - 3: Mild Pain,
4 - 6: Moderate Pain,
7 - 9: Severe Pain,
10: Worst Pain.

**Table 4:** Assessment of neck disability index (NDI) in study participants

**Assessment**	**0^th^ day**		**6^th^ session**		**12^th^ session**	
	**Score**	**Percentage**	**Score**	**Percentage**	**Score**	**Percentage**
Case 1	43	86%	11	22%	3	6%
Case 2	41	82%	18	36%	5	10%
Case 3	25	50%	17	34%	9	18%
Case 4	23	46%	14	28%	5	10%
Case 5	41	82%	17	34%	4	8%
Case 6	22	44%	10	20%	4	8%
Case 7	37	72%	13	26%	12	24%
Case 8	9	18%	7	14%	6	12%
Case 9	15	30%	12	24%	6	12%
Case 10	27	54%	18	36%	9	18%
0 - 4 points / 0 - 8%: No disability,
5 - 14 points / 10 - 28%: Mild Disability,
15 - 24 points / 30 - 48%: Moderate disability,
25 - 34 points / 50 - 64%: Severe Disability,
35 - 50 points / 70-100%: Complete Disability
